# Contextual fear memory impairment in Angelman syndrome model mice is associated with altered transcriptional responses

**DOI:** 10.1038/s41598-023-45769-x

**Published:** 2023-10-30

**Authors:** Wenyue Su, Yan Liu, Aileen Lam, Xiaoning Hao, Michel Baudry, Xiaoning Bi

**Affiliations:** 1 College of Osteopathic Medicine of the Pacific, Western University of Health Sciences, 701 E. 2nd St., Pomona, CA 91766-1854 USA; 2 College of Dental Medicine, Western University of Health Sciences, Pomona, CA 91766 USA

**Keywords:** Genetics, Neuroscience, Neurology

## Abstract

Angelman syndrome (AS) is a rare neurogenetic disorder caused by UBE3A deficiency and characterized by severe developmental delay, cognitive impairment, and motor dysfunction. In the present study, we performed RNA-seq on hippocampal samples from both wildtype (WT) and AS male mice, with or without contextual fear memory recall. There were 281 recall-associated differentially expressed genes (DEGs) in WT mice and 268 DEGs in AS mice, with 129 shared by the two genotypes. Gene ontology analysis showed that extracellular matrix and stimulation-induced response genes were prominently enriched in recall-associated DEGs in WT mice, while nuclear acid metabolism and tissue development genes were highly enriched in those from AS mice. Further analyses showed that the 129 shared DEGs belonged to nuclear acid metabolism and tissue development genes. Unique recall DEGs in WT mice were enriched in biological processes critical for synaptic plasticity and learning and memory, including the extracellular matrix network clustered around fibronectin 1 and collagens. In contrast, AS-specific DEGs were not enriched in any known pathways. These results suggest that memory recall in AS mice, while altering the transcriptome, fails to recruit memory-associated transcriptional programs, which could be responsible for the memory impairment in AS mice.

## Introduction

Angelman syndrome (AS) is a rare neurodevelopmental disorder with a prevalence of approximately 1 in 10,000 to 20,000^1–4^. AS is characterized by severe developmental delay, language and cognition deficits, motor dysfunction^3,5–9^, unusually happy demeanor and, in many AS patients, seizure activity and autism-like behavior^4,5,8^. AS is caused by deficient expression of the maternally inherited *UBE3A* gene in neurons^10–16^, since the neuronal expression of paternal *UBE3A* is silenced by a long noncoding antisense RNA transcript (*UBE3A-ATS*)^17–22^*.* Common maternal UBE3A deficiency has been attributed to four genetic etiologies^12,23^: deletions of the maternal 15q11–q13 region (class I, approximately 70% of cases), paternal uniparental disomy of chromosome 15 (class II, 5%), imprinting defects (class III, 5%), and mutations in *UBE3A* (class IV, 10%). The *UBE3A* gene encodes UBE3A ligase, also known as E6-associated protein (E6AP), the founding member of the HECT (homologous to E6AP carboxy terminus) domain-containing E3 ligase family^24^.

A few lines of transgenic mice with maternal UBE3A deficiency (AS mice) have been produced and contributed tremendously to the understanding of AS pathogenesis, as these mice exhibit several features of the human disease, including reduced brain size, abnormal electroencephalogram, learning and memory deficits, motor dysfunction^25–30^, as well as impairment in long-term potentiation (LTP) of synaptic transmission^26,28,31–33^. The identification of the mechanism underlying paternal *Ube3a* gene silencing has attracted tremendous effort to find a cure for AS through the unsilencing of the paternal *UBE3A* gene, thereby restoring UBE3A expression. Along this line, animal studies have shown that for most phenotypes, the unsilencing of the *Ube3a* gene must occur immediately after, if not before, birth. In particular, restoring UBE3A in adult AS mice rescued contextual fear memory, although it did not ameliorate motor function impairment or abnormal anxiety behaviors^34,35^. These results indicate that UBE3A plays a critical role in fear memory. To further understand the mechanism underlying the functions of UBE3A in fear memory, we performed RNA-seq on hippocampal samples from both wildtype (WT) and AS mice under control conditions and following contextual fear memory recall. Our results suggest that contextual memory recall engages different transcriptional programs in AS mice than in WT mice, which could be responsible for the recall failure.

## Results

### Memory recall further segregated hippocampal gene expression in AS mice from WT mice

**Figure 1:**
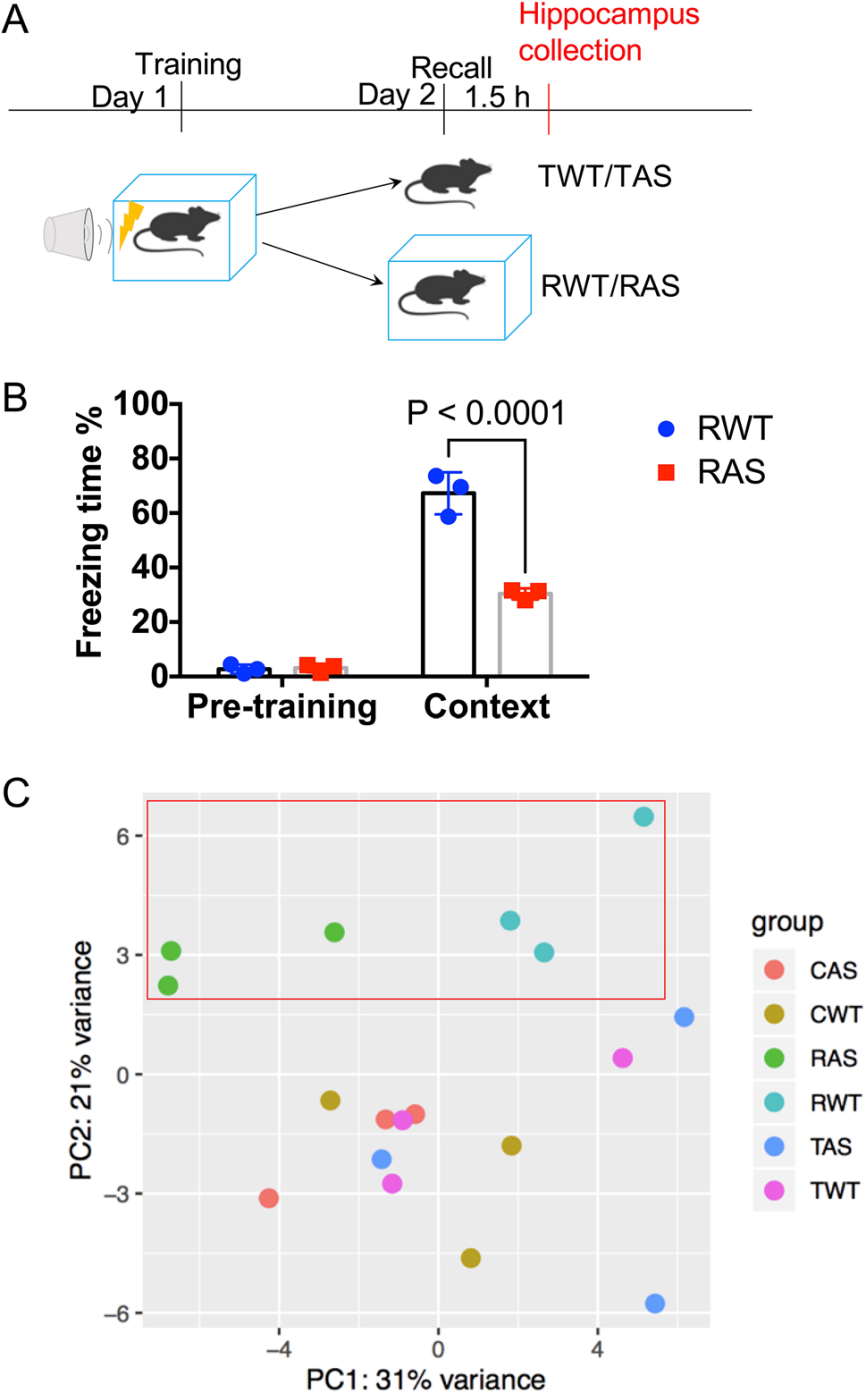
Experimental design, contextual memory recall, and overview of the transcriptome data sets. (**A**) Training and recall protocol. Trained wildtype (TWT)/trained Angelman syndrome (TAS): fear-conditioning trained WT and AS mice; recall wildtype (RWT)/recall Angelman syndrome (RAS): fear-conditioning trained and recalled WT and AS mice. (**B**) % freezing time for RWT and RAS mice in pre-training and contextual memory recall. Shown as mean ± SEM. Repeated two-way ANOVA followed by Bonferroni’s test, (Genotype x contextual memory recall) interaction: F (1, 8) = 60.91, p < 0.0001; Post hoc linear contrast: WT pre-training vs. AS pre-training, t (8) = 58.8, p > 0.9999; WT context recall vs. AS context recall, t (8) = 58.5, p < 0.0001. n = 3 mice. (**C**) Sample-to-sample principal component analysis. Each dot represents a biological replicate/mouse. PC2 scores separated samples based on behavioral test history, while PC1 separated samples by genotype. Control wildtype (CWT)/Control Angelman syndrome (CAS): home cage WT and AS mice.

To determine memory recall-induced changes in gene expression, we used 3 groups of either WT or AS mice for this study: control home-cage mice (control wildtype, CWT and control Angelman syndrome, CAS; to acquire basal gene expression in hippocampus of WT and AS mice), mice trained in the fear conditioning paradigm with no recall (trained wildtype, TWT and trained Angelman syndrome, TAS; to provide control for recall and detect long-lasting changes in gene expression induced by training), and fear conditioned mice with contextual recall at 24 h (recall wildtype, RWT and recall Angelman syndrome, RAS). Hippocampal tissues from these mice were collected at separate time points, either 24 h after training for the conditioned/training groups or 1.5 h post memory recall exposure for the Recall groups (Fig. 1A). Like previously reported^36^, there was no genotype difference in freezing time in the pre-conditioning period, while AS mice exhibited less freezing time in context-dependent memory recall (Fig. 1B), as expected from learning impairment. To examine the main sources of variance in the RNA-seq data, we first performed principal component analysis (PCA). On the second principal component, recall mice were segregated away from the rest of the mice including no-recall-Trained mice (Fig. 1C), indicating that memory recall is a driver of the variability between the samples. In addition, there was a clear separation by genotype in the Recall groups, which was driving the first component of the variability (Fig. 1C). On the other hand, there was no significant distribution separation between control home-cage mice, CWT and CAS, and no recall-Trained mice, TWT and TAS, suggesting that memory recall further differentiates the transcriptome of AS mice from WT mice.

### Differential gene expression in hippocampus of WT and AS mice with or without memory recall

**Figure 2:**
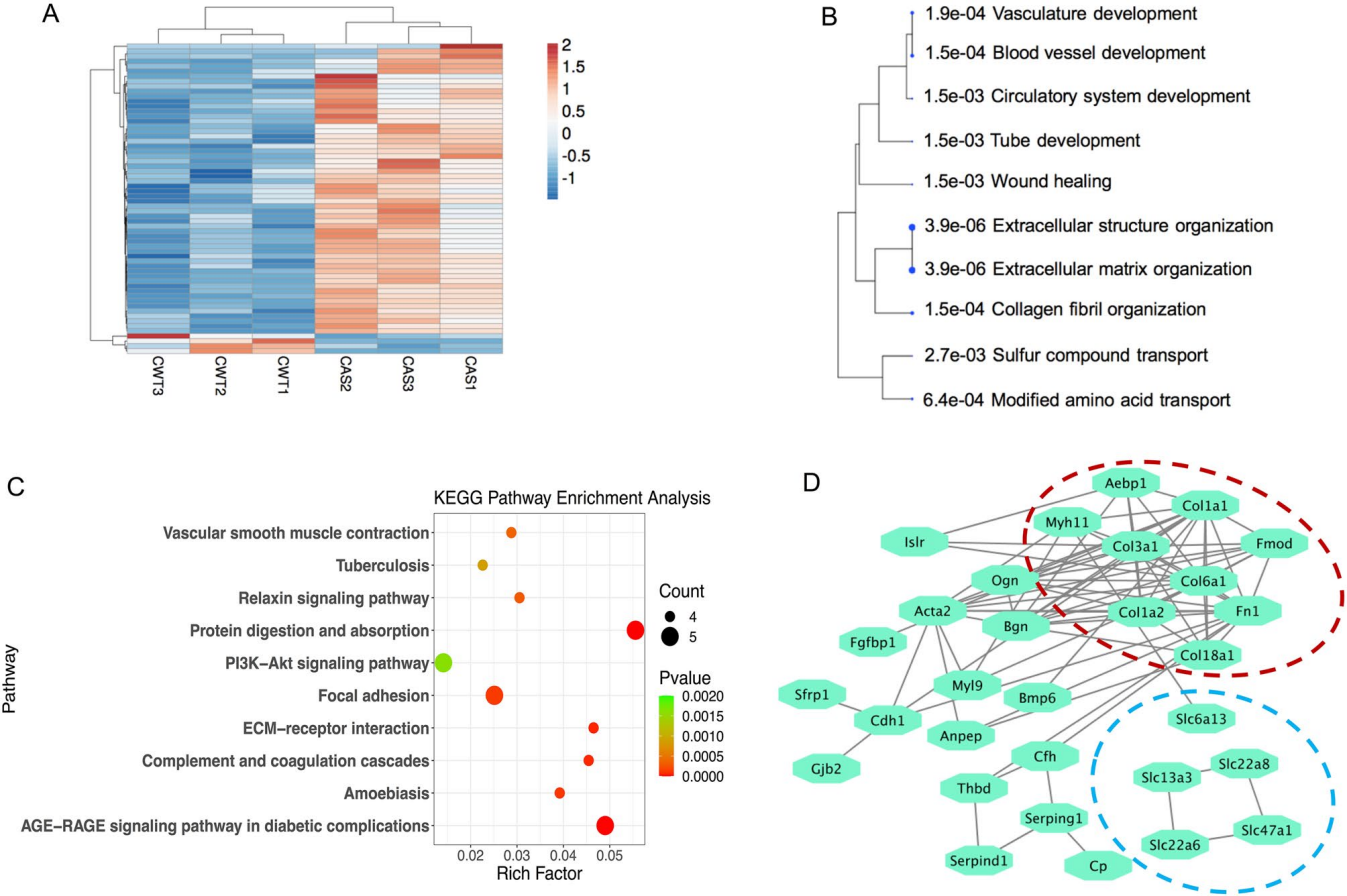
Differential gene expression analysis in hippocampus of WT and AS mice. (**A**) Heatmap showing the expression profiles of DEGs between WT and AS mice. CWT/CAS: home cage WT and AS mice. The cluster heatmap was generated using the ClustVis^37^. Each row represents an individual gene. Red color indicates increased expression and blue indicates decreased expression. (**B**) Tree view showing the 10 most significantly enriched GO (biological process) terms for DEGs. The size of the solid blue dots corresponds to the enrichment false discovery rate (FDR) with bigger dots indicating more significant changes. Terms with many shared genes are clustered together. (**C**) Top 10 enriched KEGG pathways of DEGs. Dot size indicates the number of genes annotated as participants of any KEGG pathway. Dot color corresponds to the *p*-value. (**D**) Protein association network visualization generated with STRING from DEGs. Edges represent the protein–protein associations supported by interaction sources. Dashed lines delimit protein clustering according to functional roles (see text for details).

We first compared gene expression in hippocampus from WT and AS mice under control conditions. There were 62 DEGs, with 58 genes up-regulated while only 4 genes were down-regulated in CAS, as compared to CWT mice (Fig. 2A; Supplemental Table 1). The annotation of biological processes with gene ontology (GO) indicated that most of the genes under differential regulation were related to wound healing and organ development, extracellular structure and matrix organization, and nutrient and sulfur transporters (Fig. 2B). KEGG pathway enrichment analysis showed that the pathways with the highest number of inputs included “protein digestion and absorption”, “focal adhesion,” “AGE-RAGE signaling pathway in diabetic complications”, and “PI3K-AKT pathway” (Fig. 2C).

To determine potential protein–protein interaction networks, the STRING database was searched for the 62 DEGs. As shown in Fig. 2D, the largest group of proteins with strong interactions was that involved in extracellular matrix and structure organization (circled with red dashed line). This group of proteins includes fibronectin 1(Fn1), several isoforms of collagen (Col1a1, Col1a2, Col3a1, Col6a1, Col18a1), fibromodulin (Fmod) and biglycan (Bgn), etc. It also has strong connections with osteoglycin (Ogn), actin α2 (Acta2), cadherin1 (Cdh1), and myosin (Myl9), etc. Another group of strongly connected proteins was solute carriers, including Slc13a3, slc22a6, slc22a8, and slc47a1 (circled with blue dashed line).

**Figure 3:**
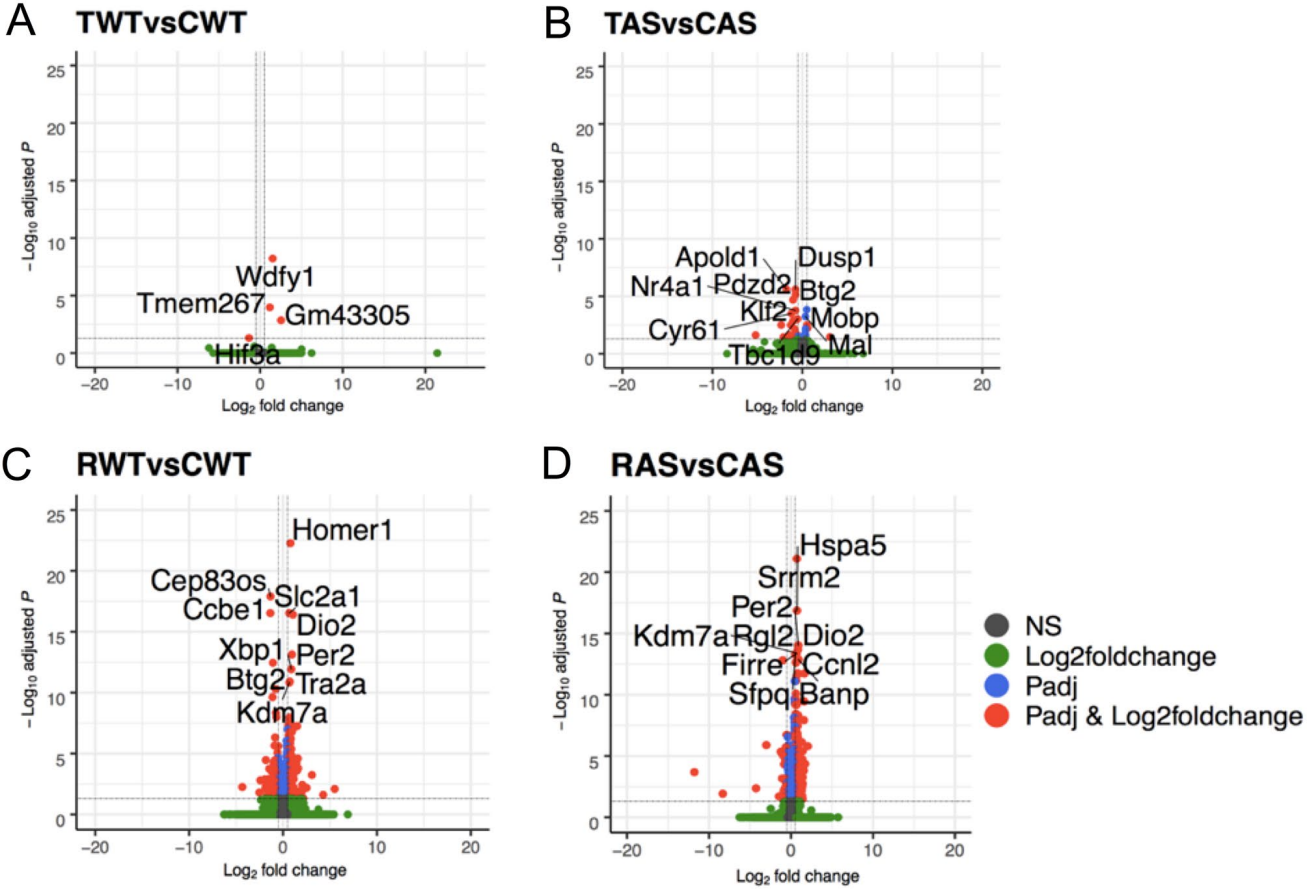
Differential gene expression analysis in hippocampus of WT and AS mice following fear conditioning and contextual memory recall. (**A**–**D**) Volcano Plot depicting up-regulated (right) and down-regulated (left) genes induced by training (**A**,**B**) or memory recall (**C**,**D**). The x-axis represents the Log2FoldChange, and the y-axis shows the − Log10 adjusted p-value. The red dots represent highly up-regulated and down-regulated genes (adjusted p-value ≤ 0.05 and a |Log_2_FoldChange|≥ 0.5). Blue dots indicate those genes with an adjusted p-value ≤ 0.05 and a |Log2FoldChange|≥ 0.1 and ≤ 0.5. Grey and green dots represent no significant change in gene expression. The top 10 DEGs with adjusted p-value ≤ 0.05 are labeled. CWT/CAS: home cage WT and AS mice; TWT/TAS: fear-conditioning trained WT and AS mice; RWT/RAS: fear-conditioning trained and recalled TW and AS mice.

We next determined DEGs induced by either fear conditioning and/or memory recall by performing pairwise comparisons. When compared to CWT, TWT mice only showed 4 DEGs 24 h after fear conditioning training. Of these, 3 genes showed higher expression and 1 showed lower expression (Fig. 3A). More DEGs were identified when TAS mice were compared to CAS; 3 genes showed higher expression and 23 genes showed lower expression (Fig. 3B). Of note, there was no overlap between DEGs identified in TWT and TAS, suggesting that fear conditioning training triggers different transcriptional programs in WT and AS mice, at least for long-lasting (24 h) changes. One possible reason for the low number of DEGs in the “Training” group is due to the fact that the samples were collected 24 h after training. By this time, expression levels of most learning-related genes have returned to basal levels^38^. The biological roles of the few long-lasting DEGs after training remain to be determined.

Similar pairwise comparison was used to assess contextual memory recall-associated changes in gene expression. When comparing RWT to CWT, there were 281 DEGs with 193 genes exhibiting higher expression after recall and 88 genes showing lower expression (Fig. 3C). Similarly, when comparing RAS mice to CAS, there were 225 genes with higher expression and 43 genes with lower expression in RAS mice (Fig. 3D). Of the identified DEGs, only 129 DEGs were shared between RWT and RAS, while the rest (152 in RWT and 139 in RAS) were genotype specific.

### Functional annotation of DEGs with GO and KEGG revealed different pathways in WT and AS mice

**Figure 4:**
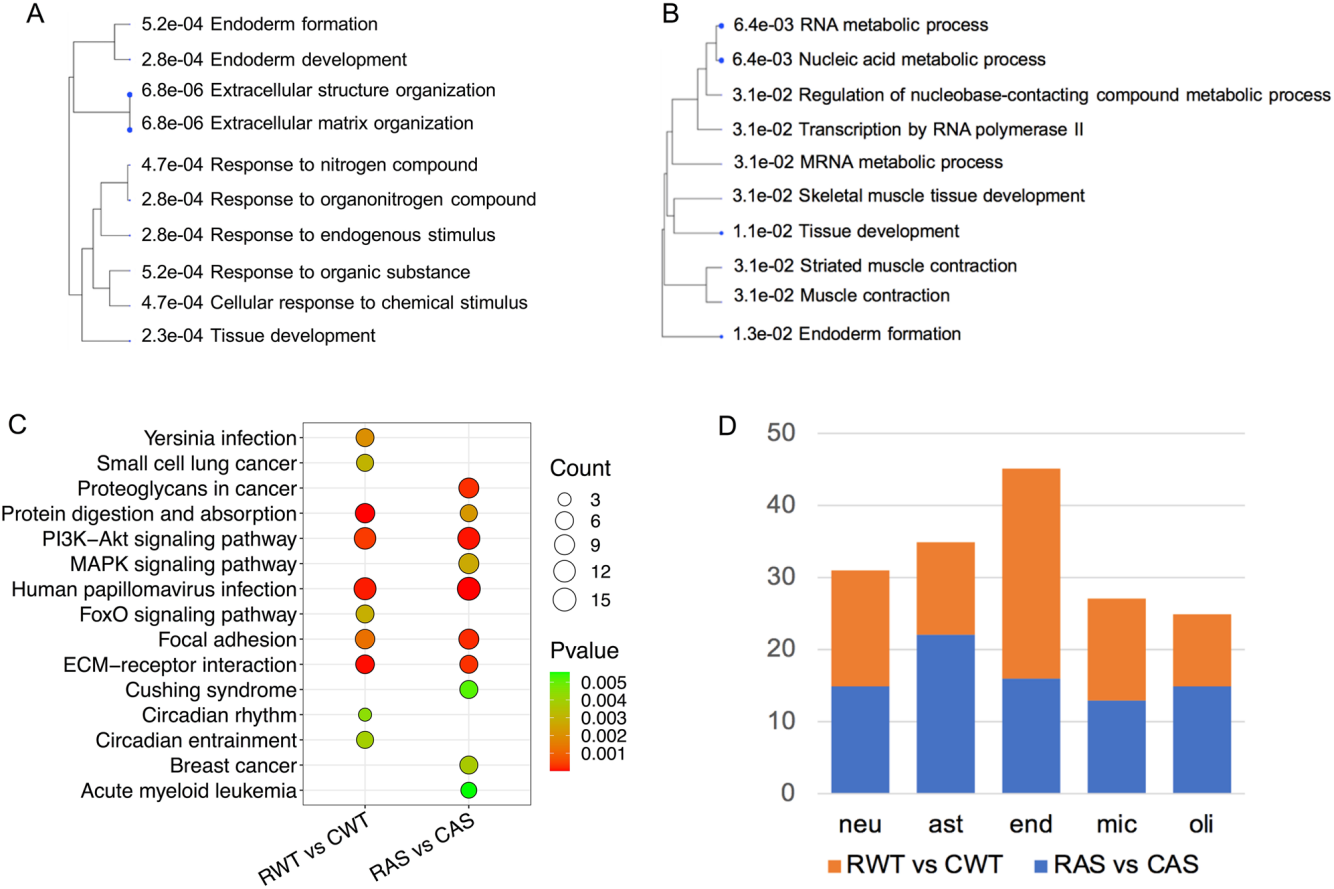
GO and KEGG pathway analysis of recall-induced DEGs in WT and AS mice. (**A**,**B**) Tree view showing the 10 most significantly enriched GO (biological process) terms for recall induced DEGs in WT (**A**) and AS (**B**) mice. Size of the solid blue dots corresponds to the enrichment FDR with bigger dots indicating more significant. Terms with many shared genes are clustered together. (**C**) Top 10 enriched KEGG pathways of DEGs. Dot size indicates the number of genes annotated as participants of any KEGG pathway. CWT/CAS: home cage WT and AS mice; RWT/RAS: fear-conditioning trained and recalled TW and AS mice. Dot color corresponds to the *p-*value. (**D**) Potential contributions of different types of brain cells (neuron, astrocyte, endothelia, microglia, oligodendrocyte) to recall induced DEGs. Cell type analysis was performed using cell type-specific markers previously identified in five purified brain cell types.

To further understand the biological function of the DEGs, we performed Gene Ontology (GO) enrichment analysis using the recall-associated DEGs. According to their false discovery rate (FDR) values, DEGs from the RWT vs CWT comparison were significantly enriched mostly in biological processes that are related to extracellular matrix organization and responses to specific signals (Fig. 4A). In contrast, the DEGs from RAS vs CAS comparison were mostly enriched in nucleic acid and RNA metabolic processes (Fig. 4B). A detailed list of genes and GO terms ranked by FDR can be seen in Supplemental Table 2.

Next, we analyzed the DEGs from recall mice compared with control mice with the KEGG enrichment, which was used to describe molecular interactions and relationship networks. KEGG analysis revealed that, among the top 10 enriched pathways, 5 were shared between WT and AS mice, including “Protein digestion and absorption”, “PI3K signaling”, “Human papillomavirus infection”, “Focal adhesion”, and “ECM-receptor interaction pathways” (Fig. 4C). Another 5 pathways were uniquely altered either in RWT or RAS. For instance, “MAPK signaling pathway” and “Proteoglycan in cancer pathway” were only found with the RAS vs CAS comparison while “FoxO signaling pathway” and “Small cell lung cancer pathway” were only found with the RWT vs CWT comparison (Fig. 4C). A detailed summary of the KEGG signaling pathways is presented in Supplemental Table 3.

We also performed cell type analysis using cell type-specific markers previously identified in five purified brain cell types^39^, namely neurons, astrocytes, endothelial cells, microglia, and oligodendrocytes. Cell type analysis revealed that the DEGs were distributed not only in neurons but also in astrocytes, endothelial cells, microglia, and oligodendrocytes. Although recall-associated DEGs in neurons were similar in WT and AS mice, AS mice had more DEGs attributed to astrocytes and WT had more to endothelial cells (Fig. 4D). These findings need to be further verified by Single-cell profiling, including spatial transcriptomics.

### Shared and distinct DEGs induced by memory recall in WT and AS mice

**Figure 5:**
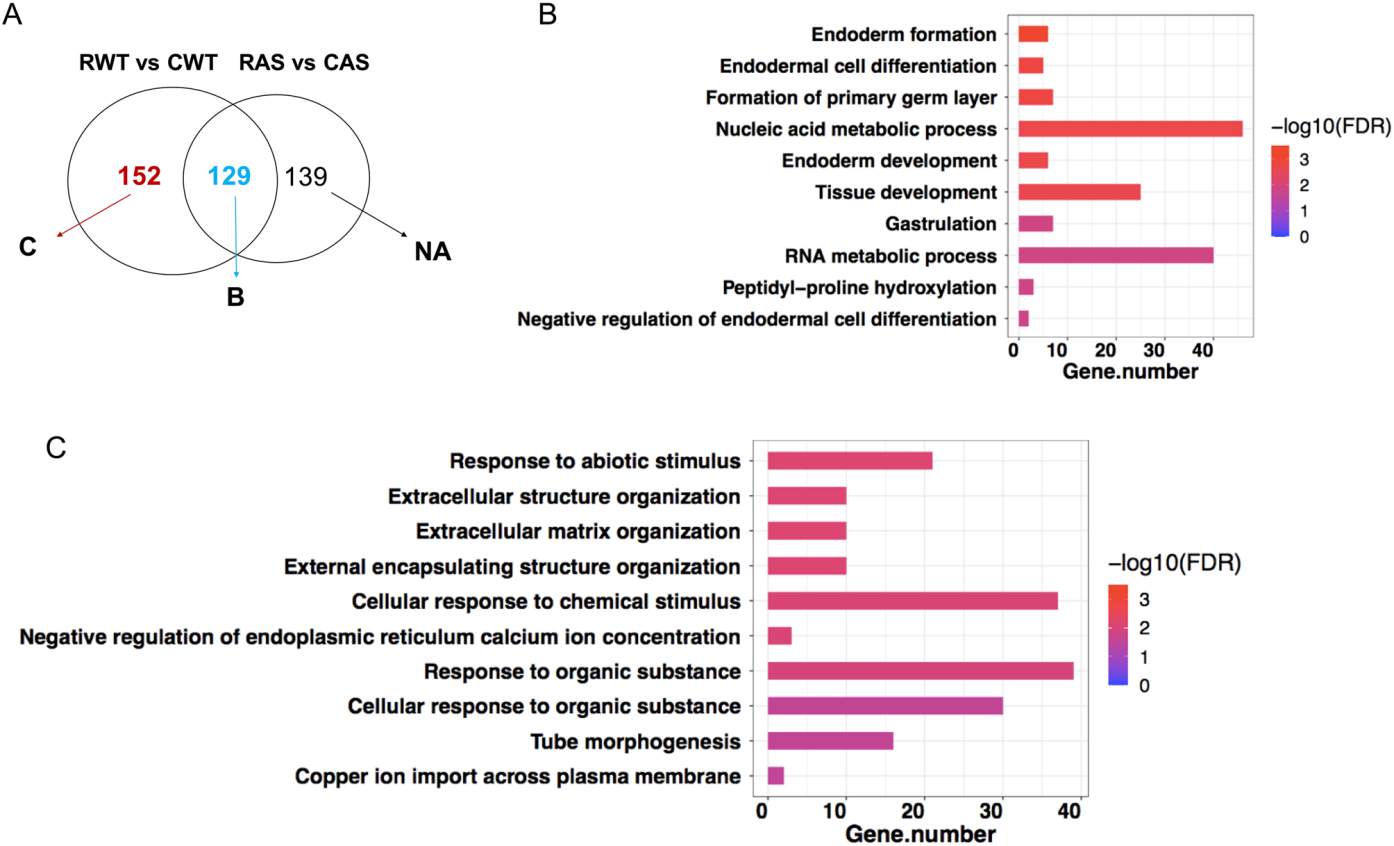
Further analysis of recall-induced transcriptomic changes in WT and AS mice. (**A**) Venn diagram (same as in Fig. 3E) showing recall-induced DEGs shared by or unique to WT and/or AS mice. CWT/CAS: home cage WT and AS mice; RWT/RAS: fear-conditioning trained and recalled TW and AS mice. The overlap of the different circles represents the number of DEGs shared by the two genotypes and the GO enrichment of which is in panel B, while that for WT unique DEGs is in C. NA (not available) indicates that AS unique recall DEGs are not enriched in any pathways. (**B**) Significantly enriched GO terms of recall-induced DEGs shared by WT and AS. (**C**) Significantly enriched GO terms of recall-induced DEGs unique to WT mice.

We next investigated in greater details the recall-related DEGs, including those shared between WT and AS mice and those specific to each genotype. Most of the 129 shared DEGs changed in the same direction with similar magnitude of changes in both genotypes (Fig. 5A, Supplemental Table 4). There were 3 exceptions: Car14 (carbonic anhydrase 14), Dnah6 (dynein axonemal heavy chain 6), and Wdr86 (WD repeat domain 86); these genes were decreased in WT and increased in AS after memory recall (Supplemental Table 4).

The top biological processes of the shared DEGs included nucleic acid and RNA metabolic processes and cell differentiation and tissue development (Fig. 5B). These DEGs most likely respond to a variety of activities. The top enriched GO terms of the 152 WT unique DEGs included cell responses to various signals, extracellular matrix, and developmental cues (Fig. 5C; Supplemental Tables 5, 6). Of note, a few genes were present across different terms, such as Sox9, Nr4a1, Col3a, and Fn1. Intriguingly, the 139 AS unique recall DEGs (Supplemental Tables 5) were not significantly enriched in any GO biological processes, indicating a non-specific/random gene activation.

**Figure 6:**
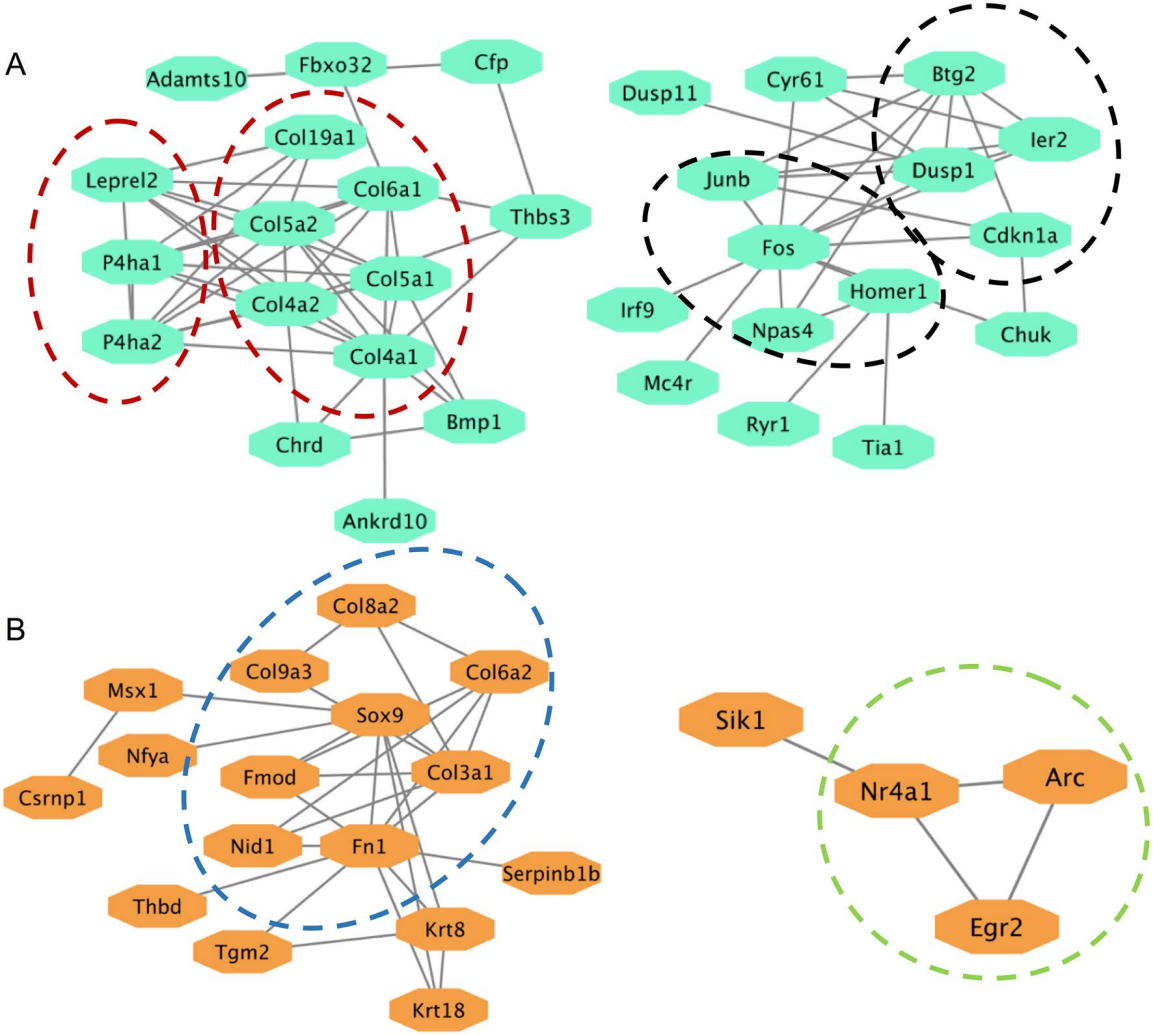
Protein network analysis. (**A**) STRING protein network analysis of the 129 shared genes. (**B**) STRING protein network analysis of 152 WT recall unique genes. Edges represent the protein–protein associations supported by interaction sources. Dashed lines delimit protein clustering according to the functional roles (see text for details).

We next performed STRING analysis of the 129 shared DEGs (Fig. 6A). An obvious cluster was the collagen family proteins and their interacting/modifying proteins, including P4ha1, P4ha2 and Leprel2 (a.k.a prolyl 3-hydroxylase 3) (circled with red dashed lines). Another major protein interaction network was found centered around transcription factors/immediate early genes (IEGs), such as Fos, Junb, Npas4, and Homer1, with strong interactions with Btg2, Ier2, and Dusp1, Cdkn1a, etc. (circled with black dashed lines).

STRING analysis of the 152 WT unique DEGs revealed a few webs with both strong and weak connections (Fig. 6B). The largest network clustered around fibronectin1, collagen proteins, and their modifying proteins (circled with blue dashed line). Of note, this web also interacts with the transcription factor SOX9. Another interesting cluster consisted of the triangle with the 3 learning and memory associated proteins, ARC, Nr4a1, and Egr2 (circled with green dashed line).

### Validation of the memory recall-induced DEGs

**Figure 7:**
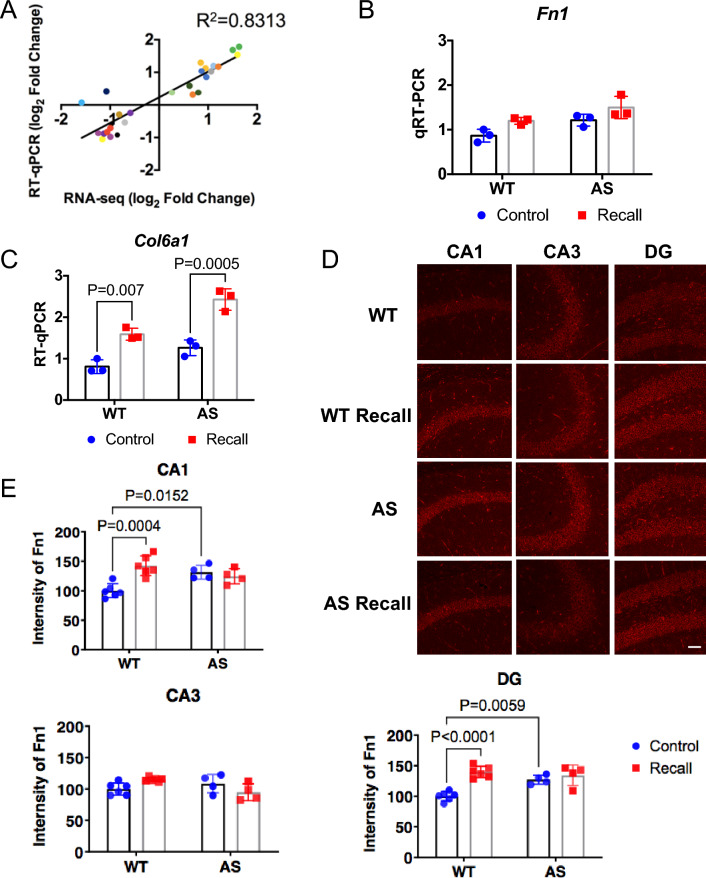
Validation of RNA-seq results. (**A**) Relation between expression levels acquired by RNA-seq and RT-qPCR, for three comparisons (CWT vs CAS, RWT vs CWT and RAS vs CAS). Means (n = 3) of selected genes were plotted as log2FoldChange value from RNA-seq and RT-qPCR. Strong Pearson correlation is shown between the expression levels measured using RNA-seq and RT-qPCR (R^2^ = 0.8313). The dots of different colors represent different genes (see Supplemental Table 7). (**B**) The expression of Fn1 was detected by RT-qPCR. Two-way ANOVA followed by Bonferroni’s test, (Genotype x contextual memory recall) interaction: F (1, 8) = 0.07372; Post hoc linear contrast: WT control vs. AS control, t (8) = 11.88, p = 0.1812; WT control vs. WT recall, t (8) = 10.69, p = 0.2204; AS control vs. AS recall, t (8) = 10.69, p = 0.4011. n = 3. (**C**) The expression of Col6a1 was detected by RT-qPCR. Two-way ANOVA followed by Bonferroni’s test, (Genotype x contextual memory recall) interaction: F (1, 8) = 2.899; Post hoc linear contrast: WT control vs. AS control, t (8) = 33.11, p = 0.1259; WT control vs. WT recall, t (8) = 74.97, p = 0.007; AS control vs. AS recall, t (8) = 74.97, p = 0.0005. n = 3. (**D**) Representative images of Fn1 immunostaining in hippocampus of control WT and AS (home cage), WT Recall and AS Recall mice. Images were acquired with a 20 X objective. Scale bar, 50 µm. (**E**) Quantification of Fn1 immunofluorescence intensity in hippocampal subregions (CA1, CA3, and DG). Shown are means ± SEM. CA1, (Genotype x contextual memory recall) interaction: F (1, 16) = 15.63, p = 0.0011; Post hoc linear contrast: WT control vs. AS control, t (16) = 1.215, p = 0.0152; WT control vs. WT recall, t (16) = 8.007, p = 0.0004; AS control vs. AS recall, t (16) = 8.007, p > 0.9999. CA3, (Genotype x contextual memory recall) interaction: F (1, 16) = 9.372, p = 0.0075; Post hoc linear contrast: WT control vs. AS control, t (16) = 1.608, p > 0.9999; WT control vs. WT recall, t (16) = 0.02324, p = 0.1309; AS control vs. AS recall, t (16) = 0.02324, p = 0.4727. DG, (Genotype x contextual memory recall) interaction: F (1, 16) = 11.74, p = 0.0035; Post hoc linear contrast: WT control vs. AS control, t (16) = 5.132, p = 0.0059; WT control vs. WT recall, t (16) = 23.84, p < 0.0001; AS control vs. AS recall, t (16) = 23.84, p > 0.9999. Two-way ANOVA followed by Bonferroni’s test. WT, n = 6 mice; AS, n = 4 mice.

To validate the changes in gene expression obtained by RNA-seq, the expression of 17 genes (Supplemental Table 7), which showed distinct expression patterns in three comparisons according to RNA-seq, was analyzed by RT-qPCR. We found a strong correlation between RNA-seq and RT-qPCR data (R^2^ = 0.8313; P < 0.0001; Fig. 7A), demonstrating the reliability of the results. Since gene expression in the ECM network was differentially altered in WT vs AS mice after recall, we performed RT-qPCR on Fn1 and Col6a1. Expression of Fn1 was significantly increased in RWT mice, as compared to CWT (Fig. 7B) when compared with unpaired t-test, which is consistent with the RNA-seq data. However, it was not statistically significant when using two-way ANOVA, possibly due to the small number of animals used. Fn1 level was higher in CAS than in CWT, and higher in RAS than in RWT mice, although neither comparison reached statistical significance. Expression of Col6a1 was increased after recall in both WT and AS mice (Fig. 7C). Immunostaining showed that FN1 was expressed mostly in the pyramidal cell body and dentate granular layers in hippocampus (Fig. 7D) with the highest expression in the subiculum (Fig. S1), which is consistent with the in-situ hybridization data from Allen Brain Atlas (http://mouse.brain-map.org/experiment/show/72119593). Quantitative analysis indicated that recall significantly increased FN1 immunoreactivity in CA1, CA3, and dentate gyrus of WT mice, but not of AS mice (Fig. 7E).

## Discussion

Learning and memory impairment in the fear-conditioning paradigm has been widely reported in AS mice^26,28,33^; yet the underlying mechanism is not completely understood. Using RNA-seq, the present study showed that contextual memory recall-induced transcriptional changes in hippocampus of AS mice were significantly different from that of WT mice. In particular, principal component analysis showed that gene expression segregation between WT and AS mice was clearer after memory recall than under control condition, suggesting that UBE3A deficiency significantly modifies cellular responses to fear memory recall. Further data analyses revealed that memory recall activated distinct biological processes/pathways in WT and AS mice, although there were some shared pathways. The shared pathways with the largest DEG numbers included nucleic acid metabolic processes and tissue development, indicating that these processes are commonly activated by a variety of environmental cues. The WT unique pathways included cellular responses to various stimuli and extracellular structure organization. The functions of these pathways in synaptic plasticity and memory consolidation have been widely reported^40–45^. No significant enrichment was found in these pathways in AS mice, suggesting that the lack of UBE3A reduced their activation. No significant enrichment in GO biological process was found with the 139 AS unique recall DEGs, which again suggests a lack of an orchestrated response to fear memory recall.

Several groups, including ours, have reported reduced dendritic spine density, especially for mature spines, in the hippocampus of AS mice^33^. The extracellular matrix plays critical roles in brain development and in spine and synaptic maturation and plasticity^40,41^. ECM can influence the formation and differentiation of neurons and other cells in the brain, thereby affecting the establishment of neural circuits and the formation of memories^40,42–45^. ECM stabilizes and remodels dendritic spines by a variety of mechanisms, including structural restriction, adhesion, ligand/receptor-driven intracellular signaling and epitope unmasking by proteases^41^. Our RNA-seq study showed that the ECM ontology was highly enriched with DEGs between WT and AS mice and the expression of these genes was upregulated in AS mice. Whether such upregulated ECM gene expression maintains spines/synapses in the immature stage and prevents their maturation and function in memory consolidation is an interesting question.

Of note, 5 of the10 ECM DEGs encode for fibronectin and collagens. Our RT-qPCR and immunostaining results indicated that while recall significantly increased FN1 expression in WT mice, no similar response was observed in AS mice. FN1 is involved in several key processes during brain development, including neuronal migration, differentiation, and synapse formation^46^. In particular, FN1 has been shown to regulate the activity of various signaling pathways involved in brain development, including extracellular signal-regulated kinase (ERK) pathways^47,48^. Recently, it has been shown that irisin, an exercise-linked hormone produced by cleavage of the fibronectin type III domain containing protein 5, provides neuroprotection in a brain derived neurotrophic factor (BDNF)-dependent manner^49^. Irisin induces BDNF accumulation in hippocampal neuronal cultures and stimulates transient ERK activation thereby preventing amyloid-β oligomer-induced oxidative stress in primary hippocampal neurons^50^. We previously showed that upregulation of BDNF by treatment with an AMPA receptor modulator (ampakine), significantly improved synaptic plasticity and fear conditioning performance in AS mice^28^. It is thus conceivable that changes in ECM expression affect spine and synaptic plasticity by altering signaling from various trophic factors, hormones, and neuromodulators. On the other hand, BDNF has been shown to regulate the expression and function of ECM^51^. The altered ECM gene expression may also be linked to inappropriate BDNF signaling in AS mice.

Many of the ECM functions are through binding to integrins since fibronectin and collagens contain the tripeptide arginine-glycine-aspartic acid (RGD), which is the recognition site for adhesive binding with integrins^52,53^. RGD peptides have been shown to evoke changes in synaptic plasticity and structural stability^54–58^, suggesting that the RGD-containing proteins found in our study (Fn1, Col18a1, Col1a1, Col3a1, Col1a2, Col6a1) may be involved in synaptic plasticity. Whether abnormal RGD-mediated integrin signaling also contributes to spine pathology in AS remains to be determined.

Another prominent DEG, which is also a FN1 interacting partner, is Col6a1 of collagen type VI (ColVI). Recent studies have shown that ColVI proteins assemble into a unique supermolecular structure to form the characteristic beaded collagen microfilaments present in ECM^59^. This super structure enables its interactions with not only other ECM filaments but also various signaling molecules and membrane-localized receptors and channels through which it regulates multiple cellular functions, such as mitochondrial integrity, autophagy, cell differentiation and survival, and tumor growth and migration^59–63^. Genetic mutations of *COL6A1-6* or their abnormal expressions have been linked to various diseases, including muscular dystrophies^64,65^. A recent study has implicated ColVI in Alzheimer’s disease (AD); in this case, both mRNA and protein levels of *Col6a1* were increased in hippocampus of a mouse model of AD^66^. Furthermore, deletion of *Col6a1* enhanced ß-amyloid toxicity, while treatment with ColVI prevented ß-amyloid-induced cell death^66^. Another recent study has linked *COL6A2* gene mutations to progressive myoclonus epilepsy^67^, while polymorphisms of several *COL6A* genes are identified as rare risk factors for schizophrenia and bipolar disorders^68^. A recent behavioral study showed that *Col6a1* deficiency results in social memory and object recognition impairment, which is associated with decreased brain dopamine and 5-HT levels^69^. It is thus conceivable that changes in collagen gene expression may contribute to abnormal spine/synapse maturation, circuitry wiring, and cognitive functions in AS.

Besides ECM, some other pathways may also contribute to AS pathogenesis. We noted higher expression in RAS mice of the Dimt1 (DIM1 dimethyladenosine transferase 1-like) gene coding for a methyltransferase that is involved in protein translation and is essential for ribosome biogenesis^70^, thus regulating cell proliferation and growth^71^. We also observed upregulation of the Sox18 gene and downregulation of the Sox9 gene in WT recall; both are components of the SOX transcription factor group involved in the regulation of embryonic and adult neurogenesis^72^. It has been reported that Sox9 downregulation is required for neurogenesis^73^, whereas the Sox18 is significantly downregulated in hippocampus of mice exhibiting cognitive impairment following sevoflurane exposure, suggesting a potential role of Sox18 in cognition^74^. Thus, changes of Sox9 and Sox18 in the WT Recall group could be related to normal learning ability, while the lack of similar changes in AS mice may contribute to memory deficits.

As mentioned earlier, principal component analysis showed that gene expression segregation between WT and AS mice was clearer after memory recall than under control condition. One interpretation of these results could be that fear-conditioning learning in AS mice failed to trigger changes in expression of genes that are essential for memory formation. Along this line, we previously showed that stronger or repetitive stimulations could rescue synaptic plasticity impairment in AS mice when a single stimulation failed^75^. Likewise, treatment with an ampakine, which enhances AMPA receptor function, or a SK2 potassium channel blocker, which facilitates NMDA receptor opening, also rescued long-term potentiation and memory impairment in fear-conditioning in AS mice^28,29^. It is thus tempting to speculate that the fear-conditioning training used in the current study, while appropriate to trigger the biological processes leading to memory coding in WT mice, was not sufficient in AS mice.

In conclusion, our results indicate that memory recall in WT and AS mice activates multiple transcriptional programs. Some pathways are shared by both genotypes, and most likely represent common transcriptional activities in response to various activities. The pathways specifically found in WT mice, including the ECM network clustered around fibronectin 1 and collagens, and pathways involved in responses to various stimuli, most likely play important roles in memory encoding. In contrast, the lack of such an orchestrated activation in AS mice may be one of the reasons for their memory deficits. Additionally, whether the AS unique DEGs, which are not enriched in any known biological processes, simply create “noise” background, or further hinder memory encoding remains to be determined.

One potential weakness is the small number of animals used in this study. Future research with a larger number of animals may further expand our findings.

## Methods

### Mice

Animal procedures were approved by the Institutional Animal Care and Use Committee of Western University of Health Sciences and were conducted in accordance with the guidelines of the NIH. Experiments were performed in 2–4 months old male wildtype (WT) and UBE3A-deficient (AS) mice housed in a 12-h light/dark cycle with food and water ad libitum. Original AS mice were obtained from the Jackson Laboratory, strain B6.129S7-*Ube3a*^*tm1Alb*^/J (stock no. 016590), and a breeding colony was established as previously described^28^. For RNA-seq, a total of 18 mice were used (3/group).

### Fear conditioning

Fear conditioning was performed as previously described^33^. Briefly, WT and AS mice were handled for 5 days before being subjected to contextual fear conditioning. Mice were placed in the fear conditioning chamber (H10-11 M-TC, Coulbourn Instruments). After a 2 min exploration period, three tone-footshock pairings separated by 1 min intervals were delivered. The 85 dB 2 kHz tone lasted 30 s and co-terminated with a footshock of 0.75 mA and 2 s. Mice remained in the training chamber for another 30 s before being returned to home cages. Contextual memory recall was tested 1 day after training in the original conditioning chamber with 5 min recording.

### Tissue collection

Ninety minutes after recall, mice were anesthetized using isoflurane and were decapitated, and the hippocampus was rapidly dissected from the brain. Bilateral hippocampi were combined and flash-frozen in liquid nitrogen. The tissues were then transferred to a − 80 °C freezer before RNA extraction.

### RNA extraction and sequencing

Total RNA was extracted from dissected tissue using RNeasy Mini Kit (QIAGEN) and genomic DNA was removed with RNase-Free DNase Set (QIAGEN) following the manufacturer’s instruction. Total RNA concentration and quality were determined using a NanoDrop 2000 spectrophotometer (ThermoFisher Scientific), and integrity was evaluated on 1% agarose gels.

A total of 1 μg RNA was used as input for RNA sample preparation. The sequencing libraries were constructed by using NEBNext Ultra™ RNA Library Prep Kit following the manufacturer’s protocol (New England Biolabs), and sequenced at Novogene Corporation (Beijing, China).

### Sequencing data processing and bio-informatics analysis

Sequenced reads were trimmed for adapter sequences and by removal of low quality reads, and then mapped to the mouse mm10 genome version using STAR. Gene expression was then estimated by using FeatureCounts to compute read counts. To identify altered gene expression due to either genotype or fear conditioning, we performed differential gene expression analysis using DEseq2. The genes with false discovery rate (FDR) ≤ 0.05 and log2foldchange ≥ 0.5 (up-regulated genes) and log2foldchange ≤ − 0.5 (down-regulated genes) were considered as significantly differentially expressed genes (DEGs). Genes for which fold changes did not reach this threshold were not included in the functional analysis. For the functional analysis, we focused on genes with relatively high expression levels with a normalized read count of 100 or more in at least one of the groups (3 biological replicates). Enrichment in GO and KEGG pathways (https://www.kegg.jp/kegg/kegg1.html) of the selected DEGs was analyzed using ShinyGO v0.741^76^ and KOBAS tools^77–80^, respectively. The FDR ≤ 0.05 was used as the threshold to identify significant functional categories and metabolic pathways. Protein–protein interaction analysis of DEGs was based on the STRING database^81^.

### Cell type composition analysis

Taking advantage of recently reported cell type specific genes^39^, we estimated specific cell type contributions to the DEGs, using genes specific to neurons, astrocytes, microglia, endothelial cells, oligodendrocytes, and oligodendrocyte precursor cells.

### RT-qPCR

Reverse transcription was performed with 1 μg of total RNA using the High-Capacity cDNA Reverse Transcription Kit (Thermo Fisher Scientific). Quantitative PCR was performed using Fast SYBR Green Master Mix (Thermo Fisher Scientific) on a CFX 96 Real-time thermocycler (Biorad). *Gapdh* gene was used as a reference gene for 2^–ΔΔCt^ quantification. Primer sequences are listed in Supplemental Table 7.

### Immunofluorescence

Immunofluorescence was performed as preciously described^82^. Briefly, sections were blocked in 0.1 M PBS containing 10% goat serum and 0.3% Triton X-100, and then incubated in primary antibody rabbit anti-Fibronectin (1:100, ab2413, Abcam) at 4 °C. Sections were washed three times in PBS and incubated in Alexa Fluor 594 goat anti-Rabbit IgG (A-11037, Life Technologies) for 2 h at room temperature. Sections were washed again three times in PBS before being mounted onto glass slides using VECTASHIELD mounting medium with DAPI (Vector Laboratories). To ensure similar antibody exposure, brains sections from different experimental groups were processed simultaneously, including using the same antibody solution with the same incubation time and conditions. Mean fluorescence intensity was calculated in pyramidal layer and the granular cell body layer by drawing a box along the cell body layer in the same anatomic locations across different sections of different animals in all experimental groups. In each region (CA1, CA3, DG), the mean intensity of three areas was used as a measurement of Fn1 staining intensity.

### Statistical analyses

Results are reported as means ± SEM and compared using two-way ANOVA followed by Bonferroni’s test (GraphPad Prism 6) or Student’s *t* test (for pairwise comparison of RNA-seq data); statistical significance was set at *p* < 0.05, n represents the number of animals.

The study is reported under the guidance of ARRIVE guidelines.

### Supplementary Information

Supplementary Information.

## Data Availability

The sequence data were deposited in NCBI BioProject under accession number PRJNA938103. Here is the link: https://www.ncbi.nlm.nih.gov/bioproject/?term=PRJNA938103.
